# Rate of re-positive RT-PCR test among patients recovered from COVID-19

**DOI:** 10.11613/BM.2020.030401

**Published:** 2020-08-05

**Authors:** Parham Habibzadeh, Mohammad M. Sajadi, Amir Emami, Mohammad Hossein Karimi, Mahboobeh Yadollahie, Maryam Kucheki, Sahar Akbarpoor, Farrokh Habibzadeh

**Affiliations:** 1 Persian BayanGene Research and Training Center, Shiraz University of Medical Sciences, Shiraz, Iran; 2 Institute of Human Virology, University of Maryland School of Medicine, Baltimore, United States of America; 3 Burn and Wound Healing Research Center, Microbiology Department, Shiraz University of Medical Sciences, Shiraz, Iran; 4 Transplant Research Center, Shiraz University of Medical Sciences, Shiraz, Iran; 5 R&D Headquarters, Petroleum Industry Health Organization, Shiraz, Iran; 6 Department of Nursing, Larestan University of Medical Sciences, Larestan, Iran

**Keywords:** COVID-19, RT-PCR, laboratory test

Reverse transcription polymerase chain reaction (RT-PCR) test is the most important laboratory test we currently rely on for the diagnosis of Coronavirus disease 2019 (COVID-19). There is no effective treatment or vaccine for COVID-19 (*1*). One option proposed to save lives is to use convalescent plasma obtained from recovered patients (*2*). There are reports that recovered patients may carry the whole virus and/or viral ribonucleic acid (RNA) (RT-PCR-positive) for long periods (*3*, *4*).

Recently, health care officials in Larestan (Iran) decided to use the convalescent plasma to treat newly infected patients. They asked 35 patients with confirmed COVID-19 who had been hospitalized in Larestan Hospital during March 22 to March 26, 2020, to donate plasma. The diagnosis was made by RT-PCR according to a method described earlier (*5*). The RNA was extracted from nasopharyngeal swabs (Invitrogen ChargeSwitch Total RNA Cell Kit, Invitrogen Co, USA). The assay tested the *E* and *RdRP* genes (*5*). Both internal and negative controls were performed for tests. Thirteen of the 35 invited patients did agree to donate. They were thus re-tested with RT-PCR (using the same method) to determine if they were RT-PCR-negative. The study protocol was approved by the Petroleum Industry Health Organization Institutional Review Board.

Reverse transcription polymerase chain reaction test was positive in 9 (5 male, 4 female) of 13 recovered patients. They had a median age of 52 (interquartile range (IQR) 30 to 56) years. They presented mostly with cough, fever, malaise, and dyspnoea. On admission, 6 had abnormal findings in their chest and mediastinal unenhanced spiral computed tomography, including ground-glass opacities with or without consolidation; 4 had a positive C-reactive protein test; and 5 had 1 or 2 underlying medical conditions, most commonly hypertension (4 of 5). The disease severity was mild to moderate. The patients were hospitalized for a median of 5 (IQR 3 to 10) days. During hospitalization, they received osteltamivir (75 mg taken orally every 12 hours) and/or lopinavir/ritonavir (400/100 mg taken orally every 12 hours). They were discharged home when their symptoms resolved completely. Other laboratory parameters measured at the time of admission were within normal limits (Table 1). The second RT-PCR test was found positive in these patients after a median of 29 (range 22 to 54) days after initiation of their symptoms/illness and 18 (range 15 to 48) days after complete resolution of their symptoms.

**Table 1:** Baseline characteristics at hospital admission in 9 studied patients

**Parameter**	**Median (IQR)**
Body mass index (kg/m^2^)	26.5 (22.8–29.0)
Haemoglobin (g/L)	141 (129–168)
White blood cell count (x10^9^/L)	4.4 (3.9–5.3)
Absolute lymphocyte count (x10^9^/L)	1.77 (1.38–2.17)
Platelet (x10^9^/L)	172 (129–201)
Serum creatinine (μmol/L)	88 (88–115)
Serum sodium (mmol/L)	143 (139–145)
Serum potassium (mmol/L)	3.5 (3.3–4.0)
Serum bicarbonate (mmol/L)	23.8 (22.7–25.4)
Arterial pH	7.39 (7.34–7.42)
Arterial pCO_2_ (kPa)	5.5 (5.1–5.9)
IQR – interquartile range.	

Observing 9 positive RT-PCR tests in 13 recovered patients after a median of 18 days of complete resolution of their symptoms – a positive rate of almost 70% – is very high. Even if we assume that all the remaining invited (but unattended) 22 recovered patients would have tested negative, the rate was still high – 26% (95% confidence interval (CI): 11% to 41%). Considering the low sensitivity (high false-negative rate) of RT-PCR test for the diagnosis of COVID-19 in nasopharyngeal samples, this rate would clearly be an underestimation (*6*). On account of the low sample size studied, the results might be considered trivial, at the first glance; however, even the lower limit of the 95% CI of the rate, 11%, is unacceptably high and potentially dangerous.

This high RT-PCR re-positive rate would have serious health implications in the world and might even change our strategies to tackle with the current pandemic. These patients, although asymptomatic, can potentially spread the virus after more than 2 weeks (even 48 days) of complete resolution of their symptoms. It is, however, worth to note that RT-PCR does not discriminate between intact whole virus and viral RNA. Therefore, a positive test does not necessarily imply an active infection or ability to transmit infection. This underlines the importance of developing tests to detect active viral replication and employing an active surveillance for identifying those infected with the virus, even asymptomatic people.
